# Genetic Risk Scores Associated with Baseline Lipoprotein Subfraction Concentrations Do Not Associate with Their Responses to Fenofibrate

**DOI:** 10.3390/biology3030536

**Published:** 2014-08-25

**Authors:** Alexis C. Frazier-Wood, Mary K. Wojczynski, Ingrid B. Borecki, Paul N. Hopkins, Chao-Qiang Lai, Jose M. Ordovas, Robert J. Straka, Micheal Y. Tsai, Hemant K. Tiwari, Donna K. Arnett

**Affiliations:** 1 USDA/ARS Children’s Nutrition Research Center, Baylor College of Medicine, Houston, TX 77030, USA; 2 Department of Genetics, Washington University School of Medicine, St. Louis, MO 63110, USA; E-Mails: mwojczyn@dsgmail.wustl.edu (M.K.W.); iborecki@wustl.edu (I.B.B.); 3 Department of Internal Medicine, University of Utah, Salt Lake City, UT 84132, USA; E-Mail: paul.hopkins@utah.edu; 4 Nutrition and Genomics Laboratory, Jean Mayer-US Department of Agriculture Human Nutrition Research Center on Aging, Tufts University, Boston, MA 02111, USA; E-Mails: chao.lai@tufts.edu (C.Q.L.); jordov01@tufts.edu (J.M.O.); 5 The Department of Epidemiology and Population Genetics. Centro Nacional Investigación Cardiovasculares (CNIC), Madrid 28029, Spain; 6 IMDEA Food, Madrid 28049, Spain; 7 Department of Experimental and Clinical Pharmacology, College of Pharmacy, University of Minnesota, Minneapolis, MN 55455, USA; E-Mail: strak001@umn.edu; 8 Department of Laboratory Medicine and Pathology, University of Minnesota, MN55455, USA; E-Mail: tsaix001@umn.edu; 9 Section on Statistical Genetics, University of Alabama at Birmingham, School of Public Health, AL 35294, USA; E-Mail: htiwari@uab.edu; 10 Department of Epidemiology, University of Alabama at Birmingham, School of Public Health, AL 35294, USA; E-Mail: arnett@uab.edu

**Keywords:** pharmacogenetics, candidate gene study, lipoprotein, fenofibrate, NMR, GOLDN, genetic risk score, particle size, LDL size, HDL size

## Abstract

Lipoprotein subclass concentrations are modifiable markers of cardiovascular disease risk. Fenofibrate is known to show beneficial effects on lipoprotein subclasses, but little is known about the role of genetics in mediating the responses of lipoprotein subclasses to fenofibrate. A recent genomewide association study (GWAS) associated several single nucleotide polymorphisms (SNPs) with lipoprotein measures, and validated these associations in two independent populations. We used this information to construct genetic risk scores (GRSs) for fasting lipoprotein measures at baseline (pre-fenofibrate), and aimed to examine whether these GRSs also associated with the responses of lipoproteins to fenofibrate. Fourteen lipoprotein subclass measures were assayed in 817 men and women before and after a three week fenofibrate trial. We set significance at a Bonferroni corrected alpha <0.05 (*p* < 0.004). Twelve subclass measures changed with fenofibrate administration (each *p* = 0.003 to <0.0001). Mixed linear models which controlled for age, sex, body mass index (BMI), smoking status, pedigree and study-center, revealed that GRSs were associated with eight baseline lipoprotein measures (*p* < 0.004), however no GRS was associated with fenofibrate response. These results suggest that the mechanisms for changes in lipoprotein subclass concentrations with fenofibrate treatment are not mediated by the genetic risk for fasting levels.

## 1. Introduction

Lipoproteins within the given fractions of very low-density, low-density, intermediate-density and high-density lipoproteins (VLDL, LDL, IDL and HDL respectively) can be further subdivided into small, medium and large subfractions based on size, which partially reflects the lipid and apolipoprotein content of the particle. Nuclear Magnetic Resonance Spectroscopy (NMR) is a method of characterizing lipoproteins in this manner, and can give information on the concentration of lipoproteins within a subfraction, as well as the average particle diameter within a fraction which reflects the constituent distribution of subfractions [1].

Specific distributions of lipoprotein subfractions, characterized by small LDL and large VLDL and HDL particles, demonstrate associations with disease states such as insulin resistance and atherosclerosis [2,3,4,5,6,7]. While the clinical utility of subfraction determination is still debated [8], the genetic association studies of the various NMR measures have shown promise as a method for helping to characterize the genetic underpinnings of lipoproteins. Several validated genetic markers have been associated with lipoprotein subclass concentrations and diameter measures in the fasting or postprandial states [9,10,11]. Although NMR measures are currently more expensive to collect than traditional enzymatic lipid measures, future clinical interest in lipoprotein subfractions may lie in the observation that subclass concentrations and distributions are modifiable, changing in response to exercise, diet and pharmacological interventions aimed at reducing atherosclerosis risk [12,13,14,15,16]. Fenofibrate is one such therapeutic agent. Indicated in hypertryglceridemia, fenofibrate reduces plasma triglyceride (TG) levels by 35%–50%, while having simultaneous effects on other parameters such as markers of inflammation, cholesterol, and lipoprotein subclasses [17,18,19,20,21,22]. In particular, fenofibrate treatment is known to shift the LDL subclass towards larger LDL particles [14,18,23]. The effects on other lipoprotein subclasses are less well established, although a few human and mice studies suggest that overall VLDL size may decrease while HDL size and number may increase [24,25,26]. Responses to fenofibrate of traditional lipid phenotypes vary, some of which can be attributed to genetic effects [27,28,29]. As yet, we are aware of only two investigations into which genetic variants may associate with differences in lipoprotein subclass changes: a recent genome-wide association study (GWAS) reported that variants in the *AHCYL2* reached and in the *CD36* genes closely approached genome-wide levels of significance with VLDL and HDL diameter responses to fenofibrate, but was limited in its ability to only detect associations of relatively large effect [30]. A further study reported that the effect of fenofibrate on HDL subclasses may be dependent on ABCA1 polymorphisms, although these remain unreplicated [26]. This study further aimed to reveal associations between genetic variants with changes in lipoprotein subfraction concentrations and distributions.

Using recently reported results from a large genome-wide association study meta-analysis for fasting NMR measures, we sought to examine whether these previously validated SNP-phenotype associations with fasting NMR measures also associate with the response of NMR measures to fenofibrate. First, we combined the SNPs into a GRSs relevant to each lipoprotein measures as there are differences in the SNP-phenotype associated with each phenotype. Next we sought to confirm that such GRSs replicated with fasting measures and subsequently, determine whether the GRSs predicted the response of these phenotypes to a three-week fenofibrate trial.

## 2. Experimental Section

### 2.1. Study Population

**Figure 1:**
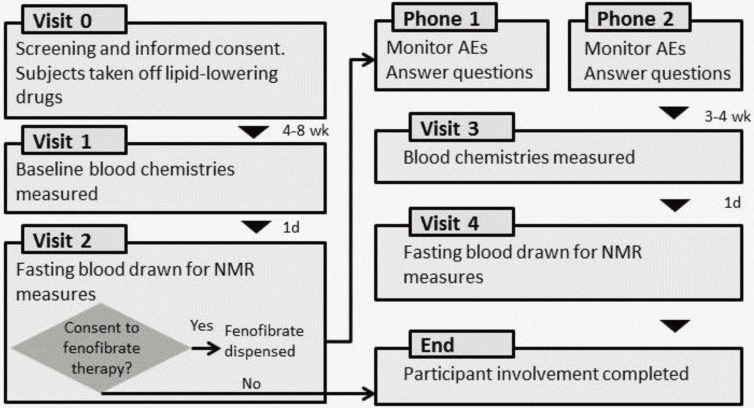
Details of the GOLDN study.

Adapted with permission from Frazier-Wood *et al.* 2012 [29].

The details of the GOLDN visits are published elsewhere [29] and depicted in Figure 1. GOLDN is part of the PROGENI (PROgram for GENetic Interaction) Network, a group of family intervention studies focusing on gene-environment interactions. The participants in the GOLDN study were mainly re-recruited from two NHLBI Family Heart Study (FHS) field centers: Minneapolis, MN, and Salt Lake City, UT. All subjects were of European ancestry. Eligibility criteria were: (1) ≥18 years of age; (2) fasting TGs <1500 mg/dL; (3) willing to participate in the study and attend the scheduled clinic exams; (4) member of a family with at least two members in a sibship; (5) AST and ALT results within normal range; and (6) creatinine ≤2.0 mg/dL. Exclusion criteria were: (1) history of liver, kidney, pancreas, gall bladder disease, or malabsorption; (2) current pregnancy; (3) insulin use; (4) use of lipid lowering drugs (including prescription, OTC and nutraceuticals; volunteers taking these agents were withdrawn from them at least three weeks prior to the study with physician’s approval); (5) use of warfarin; (6) women of childbearing potential not using an acceptable form of contraception; (7) known hypersensitivity to fenofibrate; and (8) history of pancreatitis within 12 months prior to enrollment. Previous data on these conditions were available from the parent study, and individuals not fulfilling inclusion criteria were not invited to participate. A medication questionnaire was administered on the first visit, which confirmed eligibility for inclusion. A previous study demonstrated that Caucasians in UT and MN were homogeneous and pooling data across centers would not threaten the validity of this study [31]. From an initial sample size of 1238, we evaluated 817 participants whom agreed to undergo the fenofibrate trial and are included in the analysis.

After granting informed consent, participants underwent a baseline screening visit. This visit included the collection of demographic data and smoking status (current/non). The following day, participants came to the clinic for a fasting (8-h minimum) blood draw. The fenofibrate intervention followed, and consisted of a three-week treatment period, in which participants took fenofibrate (160 mg) daily. Lipoproteins were measured again, on the last day of the treatment period after another minimum 8-h fast. The GOLDN trial also included a high-fat meal challenge, with post-prandial blood measurements taken. As these data are not included in the present analyses, this part of the GOLDN protocol is not described.

### 2.2. Biochemical Measurements

All plasma samples used for this analysis were collected after an 8-h fast. All samples were analyzed for lipoprotein and lipid profiles once all collections were made in each study.

*NMR measures* Measurements of VLDL, LDL and HDL diameter were determined by NMR spectroscopy. NMR detects the signal emitted by lipoprotein methyl-group protons when in the field of a magnet charged at 400 MHz. The NMR signal is decoded to obtain estimates of particle numbers for each of several lipoprotein fractions. The weighted average particle diameter for each lipoprotein fraction (VLDL, LDL and HDL) is calculated as the sum of the average lipoprotein particle diameters multiplied by the relative mass percentage, based on the amplitude of the methyl NMR signal and given in nanometers (nm). NMR groups intermediate-density lipoproteins (IDL) as a subclass of LDL [1,32]. The phenotypes available in GOLDN, and used in the current analyses are given in Table 1, alongside the range of diameters within each subfraction. In addition, mean diameter was available.

*Traditional lipid measures* Protocols for measuring TGs have been previously described [33,34]. Briefly, TGs were measured using the Roche/Hitachi 911 Automatic Analyzer (Roche Diagnostics Corporation, Indianapolis, IN, USA), using a glycerol blanked enzymatic method. The interlaboratory coefficients of variation in a pooled plasma control were 2.6%. Fasted TGs were available at two time points for a subsample of the current population (N = 748). The pairwise Pearson correlation between the two measures was high at *r* = 0.90 (*p* < 0.001).

**Table 1:** Diameter ranges of lipoprotein subclasses when measured by Nuclear Magnetic Resonance Spectroscopy (NMR).

NMR Lipoprotein Parameter	Diameter Range (nm)
**VLDL**	
Large VLDL/chylomicrons	>60
Medium VLDL	35–60
Small VLDL	27–35
**LDL**	
Large LDL	21.2–23
Small LDL	18–21.2
**HDL**	
Large HDL	8.8–13
Medium HDL	8.2–8.8
Small HDL	7.3–8.2

*Anthropometric and smoking measures* Clinical characteristics were taken at the study clinics at the baseline visit (Figure 1). Questionnaires were administered to collect demographic data and information on lifestyle attributes and medical history. Smoking status was collected by a question with a dichotomous outcome, which asked whether the participant was a current smoker. During this visit BMI was collected by trained research staff.

### 2.3. Genotyping

*Genotyping* Although SNPs were selected from previous literature, the data were taken from GOLDN’s available GWAS data. SNPs were genotyped using the Affymetrix Genome-Wide Human 6.0 array (Affymetrix, Santa Clara, CA, USA) and the Birdseed calling algorithm [35]. The samples were processed in two different batches by two different technicians. Call rates by plate and by technician were similar, thus, we determined that there were no significant batch effects. We used MACH software [36] to impute untyped SNPs using Human Genome Build 36, CEU population, as the reference, which created a hybrid dataset that included 2,543,887 SNPs, of which 584,029 were initially genotyped in the GOLDN population.

*Quality Control* As SNPs were selected from the available GWAS data, they were subject to the same quality control measures as our GWAS studies. For the whole GWAS data, SNPs were excluded that were monomorphic (55,530) or had a call rate <96% (82,462). Additionally, SNPs were excluded based on the number of families with Mendelian errors as follows: For minor allele frequency (MAF) ≥20%, removed if errors were present in >3 families (1486 SNPs); for 20% > MAF ≥10%, removed if errors were present in >2 families (1338 SNPs); for 10% > MAF ≥5%, removed if errors were present in >1 family (1767 SNPs); for MAF <5%, removed if any errors were present (9592 SNPs).

*SNP selection* SNPs were selected from a GWAS by Chasman and colleagues [9]. SNP-phenotype associations with NMR data in other recent studies were not included because they were not on direct NMR phenotypes, but rather latent factors created from the phenotypes [10], or because the subclass division was based on different diameter ranges than those available in GOLDN, and so it was not clear how to harmonize the phenotype in replication [11]. The initial discovery GWAS by Chasman and colleagues was conducted in 17,296 women of self-reported European ancestry forming the study population for the Women’s Genome Health Study (WGHS). Replication of signals was performed in two independent populations: The Framingham Heart Study (FHS) and the Precocious Coronary Artery Disease (PROCARDIS) study. One hundred and eighty-nine SNP-phenotype associations (which were replicated in both independent samples) pertained to phenotypes available in the GOLDN dataset (those not relating to lipoprotein subclasses e.g., ApoA1 levels were not available and so excluded from the current analysis). An additional 10 SNPs (forming 26 SNP-phenotype associations as most SNPs were associated with more than one phenotype) were not available in the GOLDN dataset, and 2 SNPs (3 SNP-phenotype associations) did not pass GOLDN quality control (as above) leaving a final selection of 160 SNP-phenotype associations included in this analysis.

### 2.4. Statistical Methods

*Phenotypes.* Fenofibrate response was defined as the ratio of pre- to post-fenofibrate values, evaluated while fasted. For both fasting, and fenofibrate response, skewed phenotypes (all except at fasting: concentration of small LDL, number of HDL particles, concentration of large HDL, concentration of small HDL, LDL diameter, and HDL diameter. For fenofibrate response: number of LDL particles, LDL diameter, and HDL diameter) were log transformed to approximate normality before analysis. To test whether there were significant differences NMR measures before and after the fenofibrate trial, paired t tests were conducted. Please check the highlights.

*SNP-phenotype associations.* We used a linear mixed model. The effects of SNP genotypes (categorical variable with three classes for genotyped SNPs; continuous variable on a scale of 0–2 for imputed SNPs) were treated as fixed effects in an additive model, and the dependencies among members within each family were treated as random effects. Age, smoking status, BMI and gender were included in the model as covariates, as in the previous GWAS [9]. Field center was treated as an additional covariate. The minor allele was the coded allele. Pre-treatment fasted SNP-phenotype associations are available in Supplementary Table S1.

In replicating work from a previous study, we chose the same covariates in the genetic association analyses. These did not include baseline triglycerides (TGs). The justification behind this approach is the evidence that the pharmacological action of fenofibrate is the same in normolipidemics as hyperlipidemics, although hyperlipidemics may show slightly stronger responses [37,38,39], and the lack of evidence to suggest that the genetic mediators of fenofibrate pathways would differ across normo- and hyper-lipidemics. However, we also present the genetic analyses stratified by TG levels (baseline fasting TG ≥150 mg/dL) noting that the results do not change according to pre-treatment TG levels (Supplementary Table S2).

*GRS-phenotype associations.* Unweighted GRSs were constructed as unweighted sums as the original GWAS meta-analysis was conducted on a population which may be described as “modest” for current GWAS and unweighted GRS maybe more robust against errors in estimating the effect sizes arising from limited sample size [39]. In addition, unweighted GRS are more suitable at reducing increased estimates of association due to population heterogeneity, population substructure, and “winner’s curse” bias [39]. For the individual SNP-phenotype associations the genotypes were rescored where necessary, based on the original direction of effect in the published meta-analytic GWAS [9] (Supplementary Table S2). As SNPs could be associated with more than one lipoprotein measure in the previous GWAS, they therefore could contribute to more than one GRS in the current analyses. For the GRS, where the original beta was negative for a given SNP-phenotype association, the genotype was rescored such that the major allele was the coded allele. Where the original beta was positive, the minor allele remained the coded allele. This resulted in genotypes being scored both ways (with the minor allele and the major allele as the coded allele) based on the particular phenotype. The direction of the original betas for each SNP-phenotype association are available in Supplementary Table S2. The GRS for each phenotype was then created by summing the alleles (genotyped SNPs) and allele dosages (imputed SNPs) for all SNPs within the previously reported SNP-phenotype associations for a given measure [9]. 

GRS was used as a continuous predictor in mixed linear models as outlined for the SNP-phenotype associations, above. The results for the GRS-phenotype associations were corrected using a Bonferroni correction, based on the number of calculated GRSs (*n* = 14). This gave rise to a *p* = 0.004 (α = 0.05/14). F-values from the mixed linear models were converted to effect sizes (cohen’s d; δ).

## 3. Results

*Changes in lipoprotein subclasses.* Participant characteristics for the GOLDN study, including the change in lipoprotein measures pre- and post-fenofibrate, are shown in Table 2. As expected, we saw a significant increase in the average size of LDL particle (*p* < 0.0001) which resulted from a decrease in the concentration of small LDL (*p* > 0.0001); the concentration of large LDL did not change (*p* = 0.19; Table 2). All VLDL subclass concentrations decreased (*p* < 0.0001) in such a manner that the overall diameter of VLDL particles remained the same (*p* = 0.14; Table 2). The small and large HDL subclass concentrations also decreased (*p* = 0.003 and *p* < 0.0001 respectively) and the medium HDL subclass concentration increased (*p* < 0.0001) yielding an overall decrease in HDL diameter (*p* < 0.0001; Table 2**)**.

*GRS-phenotype associations with NMR measures at baseline, and NMR responses to fenofibrate*. Almost all GRSs were associated with baseline NMR data (*p* < 0.05), although not all associations survived a correction for multiple testing. The exception was the association between VLDL total particles and GRS (*p* = 0.11). Nine out of 14 significant GRS-phenotype associations (concentration of small VLDL particles, concentration of medium VLDL particles, average VLDL diameter, concentration of large LDL particles, LDL total particle number, concentration of small HDL particles, concentration of medium HDL particles, concentration of large HDL particles, HDL total number of particles and HDL diameter) survived a Bonferroni correction which gave rise to an corrected *p* = 0.004 (Table 3). None of the GRS were associated with fenofibrate response after a Bonferroni correction (Table 3). As fenofibrate is indicated for use in hypertriglyceridemia GRS-phenotype associations were conducted stratified by TG levels (baseline fasting TG ≥ 150 mg/dL). Results remained the same in that neither normotriglyceridemic, nor hypertriglyceridemic samples showed significant GRS-phenotype associations with responses to fenofibrate (Supplementary Table S2). 

**Table 2:** Means (standard deviation) or percentage for demographic, anthropometric and fasting lipoprotein characteristics of the GOLDN study population.

Age, year	48.42 (16.34)		
Gender, % male	48.28		
Smoker, % current	7.79		
BMI	28.25 (5.62)		
	**Baseline**	**Post-Fenofibrate**	***p***
Small VLDL concentration, nmol/L	32.93 (21.89)	22.83 (15.83)	<0.0001
Medium VLDL concentration, nmol/L	37.49 (36.74)	18.46 (20.44)	<0.0001
Large VLDL concentration, nmol/L	3.93 (7.68)	2.09 (3.24)	<0.0001
VLDL total particles; nmol/L	74.38 (50.81)	43.41 (31.73)	<0.0001
VLDL diameter; nm	51.39 (7.86)	51.84 (8.71)	0.14
Small LDL concentration, nmol/L	925.64 (557.03)	779.49 (373.04)	<0.0001
Large LDL concentration, nmol/L	407.11 (272.91)	397.23 (201.37)	0.19
LDL total particles; nmol/L	1375.33 (437.46)	1209.35 (380.68)	<0.0001
LDL diameter; nm	20.81 (0.88)	20.90 (0.58)	<0.0001
Small HDL concentration, nmol/L	21.65 (5.54)	20.76 (6.71)	0.003
Medium HDL concentration, nmol/L	3.00 (3.61)	5.92 (4.80)	<0.0001
Large HDL concentration, nmol/L	6.32 (3.54)	5.73 (3.25)	<0.0001
HDL total particles; nmol/L	30.97 (5.63)	32.42 (5.95)	<0.0001
HDL diameter; nm	8.85 (0.45)	8.73 (0.39)	<0.0001
**Baseline fasting TG < 150 mg/dL (N = 544)**			
Small VLDL concentration, nmol/L	28.13 (15.67)	18.60 (12.60)	<0.001
Medium VLDL concentration, nmol/L	21.06 (14.68)	12.34 (11.07)	<0.001
Large VLDL concentration, nmol/L	1.39 (1.53)	0.95 (1.22)	<0.001
VLDL total particles; nmol/L	50.62 (24.01)	31.92 (19.76)	<0.001
VLDL diameter; nm	50.73 (8.20)	51.12 (9.19)	0.44
Small LDL concentration, nmol/L	705.82 (403.85)	662.38 (261.74)	<0.001
Large LDL concentration, nmol/L	478.91 (252.68)	398.55 (184.97)	<0.001
LDL total particles; nmol/L	1213.23 (364.60)	1073.37 (279.02)	<0.001
LDL diameter; nm	21.14 (0.73)	21.01 (0.53)	0.001
Small HDL concentration, nmol/L	20.53 (5.17)	19.25 (6.08)	<0.001
Medium HDL concentration, nmol/L	3.34 (3.54)	6.67 (4.47)	<0.001
Large HDL concentration, nmol/L	7.27 (2.30)	6.42 (3.30)	<0.001
HDL total particles; nmol/L	31.15 (5.18)	32.35 (5.44)	<0.001
HDL diameter; nm	8.97 (0.43)	8.83 (0.34)	<0.001
**Baseline fasting TG ≥ 150 mg/dL (N = 248)**			
Small VLDL concentration, nmol/L	43.62 (28.93)	31.97 (18.10)	<0.001
Medium VLDL concentration, nmol/L	74.12 (44.03)	31.67 (28.32)	<0.001
Large VLDL concentration, nmol/L	9.60 (11.74)	4.55 (4.60)	<0.001
VLDL total particles; nmol/L	127.38 (54.65)	68.22 (37.96)	<0.001
VLDL diameter; nm	52.84 (6.85)	53.39 (7.35)	0.09
Small LDL concentration, nmol/L	1415.81 (539.35)	1032.66 (445.66)	<0.001
Large LDL concentration, nmol/L	247.00 (247.40)	413.84 (232.50)	<0.001
LDL total particles; nmol/L	1736.81 (488.611)	1503.32 (404.81)	<0.001
LDL diameter; nm	20.07 (0.72)	20.67 (0.62)	<0.001
Small HDL concentration, nmol/L	24.14 (5.54)	24.03 (8.84)	0.36
Medium HDL concentration, nmol/L	2.23 (3.65)	4.30 (5.08)	<0.001
Small VLDL concentration, nmol/L	43.62 (28.93)	31.97 (18.10)	<0.001
Medium VLDL concentration, nmol/L	74.12 (44.03)	31.67 (28.32)	<0.001
Large VLDL concentration, nmol/L	9.60 (11.74)	4.55 (4.60)	<0.001

**Table 3:** Associations between genetic risk scores and baseline NMR measures, and between genetic risk scores and the response of NMR measures to a 3-week fenofibrate trial.

NMR Measure	Min	Max	Mean (SD)	Genetic Risk Score					
				GRS-Phenotype Associations					
				Baseline			Fenofibrate Response		
				F-Value	δ	*p*	F-Value	δ	*p*
**Full GOLDN sample (*n* = 817)**									
Small VLDL concentration	9.44	22.02	15.75 (2.11)	0.04	0.01	0.85	0.45	0.05	0.50
Medium VLDL concentration	5.26	13.65	9.79 (1.49)	3.97	0.14	0.04	0.39	0.04	0.53
Large VLDL concentration	9.16	22.49	15.87 (2.34)	0.66	0.06	0.41	1.77	0.09	0.18
VLDL total particles	1.01	8.34	5.15 (1.16)	26.37	0.36	<0.0001 *	0.50	0.05	0.48
VLDL diameter	1.00	10.09	5.32 (1.61)	2.03	0.10	<0.15	0.01	0.007	0.93
Small LDL concentration	18.00	27.99	23.25 (1.63)	8.90	0.21	0.003 *	1.65	0.09	0.20
Large LDL concentration	8.37	24.01	16.29 (2.48)	11.48	0.24	0.0007 *	0.00	0	0.99
LDL total particles	5.96	19.65	12.39 (2.16)	8.10	0.20	0.004 *	0.24	0.03	0.62
LDL diameter	0.14	7.97	3.55 (1.25)	7.04	0.19	0.008 *	0.24	0.03	0.63
Small HDL concentration	5.00	18.01	10.64 (2.06)	13.86	0.26	0.0002 *	0.02	0.01	0.87
Medium HDL concentration	7.27	17.27	12.25 (1.77)	9.11	0.21	0.0003 *	6.38	0.17	0.01
Large HDL concentration	5.98	19.05	12.26 (1.97)	12.14	0.24	<0.0005 *	0.19	0.03	0.67
HDL total particles	1.19	10.88	5.91 (1.66)	2.56	0.11	0.11	0.35	0.04	0.56
HDL diameter	6.80	20.46	13.50 (2.23)	10.05	0.22	<0.002 *	0.54	0.05	0.46

* Significant at a Bonferroni corrected α = 0.004.

## 4. Conclusions

This study examined whether the change in lipoprotein subclass concentration and distribution after a three week fenofibrate trial was associated with SNPs previously identified with fasting NMR-measured lipoprotein phenotypes. We report changes in all lipoprotein measures, with the exception of VLDL distribution and the concentration of large LDL particles, after fenofibrate treatment. GRSs, composed of previously validated SNPs, were strongly associated with most baseline NMR phenotypes, but were not predictive of the response of those phenotypes to fenofibrate.

As expected from previous literature, LDL particle size significantly increased after the fenofibrate treatment [13,14,18]. In our study, this arose from a decrease in the concentration of small LDL particles, while the concentration of large LDL particles remained the same. This study provides additional support for the notion of protective effects of fenofibrate treatment for cardiovascular disease risk, as it is the smaller, more dense LDL particles that are considered to occur with insulin resistance and convey increased atherosclerosis risk, although this conclusion must be considered alongside an understanding of any lipid response to fenofibrate which is not examined in the current analyses [2,4,7,40,41,42].

The effects of fenofibrate on other fractions of lipoprotein are less well studied. We report that the concentration of all VLDL subfractions significantly decreased, such that the overall distribution remained the same, supporting a single previous report [25]. An increase in VLDL particles, especially the large subfraction, occurs in insulin resistance [40,43] and confers increased cardiovascular disease risk [44,45]. However any reduction in cardiovascular disease risk associated with the decrease in VLDL and small LDL particles, may have to be considered against an increase in small HDL particles. Although HDL may be beneficial in preventing atherosclerosis through its role in reverse cholesterol transport, this processes is associated with the large HDL subspecies [25]. We report a significant increase in small HDL particles, suggestive of an increase in cardiovascular disease risk. However, this may be partially offset by a simultaneous increase in medium and large HDL particle concentrations as evidenced by the significant shift to an overall larger HDL diameter, even though the small subfraction shows the greatest increase.

We created GRSs from previously validated SNP-phenotype associations with NMR data [9]. With the exception of total number of VLDL particles, all GRSs showed at least a trend toward association with NMR phenotypes in that they were significant, but did not survive a stringent Bonferroni correction for multiple testing. Several phenotypes remain significant even after correction: GRS-phenotype associations held for medium VLDL concentration, VLDL diameter, large LDL concentration, LDL total particles, small medium and large HDL concentrations, HDL total particles and HDL diameter. However, we saw no significant GRS-phenotype associations with responses to fenofibrate. Whether genetic loci associated with any baseline measures also associate with the response of those measures to interventions is a largely unstudied question. Previous data from our lab indicate that for lipid measures, only 7% of baseline genetic associations also associate with fenofibrate response [46]. The current study showed no shared genetic associations with baseline and response phenotypes, which seemed to confirm the broader pattern of results from the previous study. However, we cannot exclude the possibility that our loci were less robustly validated than those employed in the lipid study, due to fewer research on lipoprotein subfractions *vs.* traditional lipids. These are the first investigations of which we are aware that address this broader question. Certainly, this study represents the first investigation focusing on NMR data. We conclude that on current evidence, different genetic loci associate with baseline and fenofibrate response lipoprotein phenotypes.

The results of this study must be considered in the light of several limitations. First, as the first such study, replication is a key issue, although we are confident that the SNPs associated with fasting NMR lipoproteins in free-living populations have been robustly validated. Second, not all SNPs from the original GWAS were available in our data, either due to our quality control measures or genotyping platform. Proxy SNPs were not available in a large number of cases, and these SNPs are excluded from analysis. However, as they are likely to only contribute a small amount of information to the overall GRS we do not believe this that would affect the overall conclusion of our study. Nonetheless, to establish the validity of the GRS in and of itself, replication is vital. Finally, as a clinical trial, we did not have the power to fully replicate all of the previous SNP-phenotype associations in baseline NMR data although our GRSs, in general, showed strong GRS-phenotype associations. For replication, we would also encourage larger samples for such, where feasible, and additionally a less conservative correction for multiple testing—here we chose the most conservative Bonferroni correction as our analyses remain the first to construct such a GRS from the previous data. This would allow a more finally nuanced analysis of individual SNP-phenotype associations for the fenofibrate responses.

Our results may still have important implications for future practice. We have shown fenofibrate to be an important modulator of many lipoprotein subclass measures. However, what modulates individual differences in the changes of these phenotypes at the genetic level, remains an unanswered question. Addressing this may one day help with understanding the pathways through which fenofibrate works in individuals, to target more effective treatment.

## Supplementary Files

Supplementary File 1
