# Supplementary material for: Genetic Risk Scores Associated with Baseline Lipoprotein Subfraction Concentrations Do Not Associate with Their Responses to Fenofibrate
Source: Biology (Basel). 2014 Aug 25;3(3):536–50. doi: 10.3390/biology3030536 (PMC4192626; doi:10.3390/biology3030536)
Supplement: Supplementary File 1 [file biology-03-00536-s001.pdf]

Supplementary Information

Table S1. Initial SNP-phenotype associations with fasting NMR data.

Rs Number	Locus	*Gene*	± [a]	MAF in GOLDN	F-Value	*p*
**Small VLDL Concentration**						
rs10889353	1p31.3	*ANGPTL3*	−	0.36	2.84	0.187
rs10903129	1p36.11	*TMEM57*	−	0.47	0.26	0.771
rs11067392	12q24.11	*MVK/MMAB*	−	0.06	0.01	0.932
rs1109166	16q22.1	*LCAT*	+	0.15	0.16	0.692
rs11122490	1q42.13	*GALNT2*	−	0.26	0.04	0.526
rs12610185	19p13.11	*CILP2/PBX4/NCAN/SF4*	−	0.08	0.01	0.932
rs1363232	5q33.3	*TIMD4/HAVCR1*	−	0.32	1.04	0.309
rs1393794	11p11.2–11p11.12	*MADD/FOLH1/NR1H3*	+	0.27	4.02	0.045
rs157580	19q13.32	*APOC1-APOE*	−	0.41	0.67	0.415
rs1800775	16q13	*CETP*	+	0.45	0	0.992
rs328	8p21.3	*MADD/FOLH1/NR1H3*	−	0.10	0.6	0.44
rs646776	1p13.3	*CELSR2/PSRC1/SPRT1*	−	0.20	1.98	0.16
rs6511720	19p13.2	*LDLR*	−	0.22	0.42	0.519
rs676210	2p24.1	*APOB*	−	0.22	6.46	0.011
**Medium VLDL Concentration**						
rs10889353	1p31.3	*ANGPTL3*	−	0.36	3.12	0.028
rs11974409	7q11.23	*MLXIPL*	−	0.26	1.41	0.235
rs1260326	2p23.3	*GCKR*	+	0.41	1.62	0.203
rs12797843	11p11.2–11p11.12	*MADD/FOLH1/NR1H3*	+	0.09	0.43	0.511
rs1363232	5q33.3	*TIMD4/HAVCR1*	−	0.32	0.53	0.467
rs2304128	19p13.11	*CILP2/PBX4/NCAN/SF4*	−	0.08	1.03	0.311
rs328	8p21.3	*MADD/FOLH1/NR1H3*	−	0.1	4.01	0.046
rs405509	19q13.32	*APOC1-APOE*	−	0.48	0.33	0.568
**Large VLDL Concentration**						
rs11974409	7q11.23	*MLXIPL*	−	0.26	1.3	0.254
rs1260326	2p23.3	*GCKR*	+	0.41	3.83	0.051
rs1363232	5q33.3	*TIMD4/HAVCR1*	−	0.32	2.73	0.099
rs2076530	6p21.32	*BTNL2*	+	0.44	0.74	0.678
**Large VLDL Concentration**						
rs2697920	11p11.2–11p11.12	*MADD/FOLH1/NR1H*	−	0.38	1.29	0.257
rs328	8p21.3	*MADD/FOLH1/NR1H3*	−	0.1	0.54	0.463
rs439401	19q13.32	*APOC1-APOE*	−	0.63	0.24	0.628
**VLDL Total Particles**						
rs10889353	1p31.3	*ANGPTL3*	−	0.36	4.82	0.008
rs1109166	16q22.1	*LCAT*	+	0.15	0.05	0.826
rs11122490	1q42.13	*GALNT2*	−	0.26	1.17	0.28

Table S1. *Cont.*

Rs Number	Locus	*Gene*	± [a]	MAF in GOLDN	F-Value	*p*
		**VLDL Total Particles**				
rs1260326	2p23.3	*GCKR*	+	0.41	4.93	0.027
rs12610185	19p13.11	*CILP2/PBX4/NCAN/SF4*	−	0.08	1.16	0.281
rs12797843	11p11.2–11p11.12	*MADD/FOLH1/NR1H3*	+	0.09	0.06	0.803
rs1363232	5q33.3	*TIMD4/HAVCR1*	−	0.32	2.72	0.099
rs1800775	16q13	*CETP*	+	0.45	0.65	0.42
rs328	8p21.3	*MADD/FOLH1/NR1H3*	−	0.1	0.414	0.042
rs439401	19q13.32	*APOC1-APOE*	−	0.63	0.14	0.711
rs646776	1p13.3	*CELSR2/PSRC1/SPRT1*	−	0.20	1.86	0.173
rs676210	2p24.1	*APOB*	−	0.22	11.46	0.001
rs714052	7q11.23	*MLXIPL*	−	0.13	7.3	0.007
		**VLDL diameter**				
rs11085270	19p13.11	*CILP2/PBX4/NCAN/SF4*	+	0.22	3.06	0.081
rs1260326	2p23.3	*GCKR*	+	0.41	0.32	0.574
rs2279804	5q33.3	*TIMD4/HAVCR1*	−	0.47	0.29	0.496
rs2306813	1q42.13	*GALNT2*	+	0.19	1.37	0.088
rs676210	2p24.1	*APOB*	+	0.22	24.71	<0.0001
		**Small LDL Concentration**				
rs10769253	11p11.2–11p11.12	*MADD/FOLH1/NR1H3*	−	0.18	2.02	0.156
rs10808546	8q24.13	*TRIB1*	−	0.45	1.88	0.1708
rs11974409	7q11.23	*MLXIPL*	−	0.26	2.88	0.09
rs1800588	15q22.1	*LIPC*	−	0.22	0.72	0.395
rs1800775	16q13	*CETP*	+	0.45	0.78	0.378
rs2271293	16q22.1	*LCAT*	−	0.12	1.16	0.173
rs331	8p21.3	*MADD/FOLH1/NR1H3*	−	0.26	0.71	0.401
rs4731702	7q32.2	*KLF14*	−	0.50	0.05	0.827
rs4846904	1q42.13		+	0.20	0.59	0.4435
rs6065906	20q13.12.B	*PLTP*	+	0.15	0.36	0.551
rs646776	1p13.3	*CELSR2/PSRC1/SPRT1*	−	0.20	13.12	0.0003
rs7307277	12q24.31.B	*CCDC92/DNAH10/ZNF664*	−	0.33	0.54	0.381
rs780094	2p23.3	*GCKR*	+	0.40	4.48	0.083
		**Large LDL Concentration**				
rs1535	11q12.2	*FADS1-3*	−	0.36	5.98	0.015
rs1713222	2p24.1	*APOB*	−	0.16	1.3	0.234
rs1800588	15q22.1	*LIPC*	+	0.22.	3.77	0.053
rs1864163	16q13	*CETP*	−	0.24	2.64	0.105
rs2083637	8p21.3	*MADD/FOLH1/NR1H3*	+	0.27	4.44	0.036
rs2271293	16q22.1	*LCAT*	+	0.12	0.78	0.257
rs2957873	11p11.2–11p11.12	*MADD/FOLH1/NR1H3*	+	0.20	0.83	0.362

Table S1. *Cont.*

Rs Number	Locus	*Gene*	± [a]	MAF in GOLDN	F-Value	*p*
			**Large LDL Concentration**			
rs4803750	19q13.32	*APOC1-APOE*	−	0.07	0.86	0.196
rs4808206	19p13.11	*CILP2/PBX4/NCAN/SF4*	+	0.16	0	0.978
rs4810479	20q13.12.B	*PLTP*	−	0.21	0.01	0.927
rs4846904	1q42.13	*GALNT2*	−	0.20	1.78	0.182
rs4939883	18q21.1	*LIPG*	−	0.21	0.78	0.283
rs5744680	5q13.3	*HMGCR*	+	0.39	1.82	0.258
rs646776	1p13.3	*CELSR2/PSRC1/SPRT1*	−	0.20	2.09	0.149
rs6511720	19p13.2	*LDLR*	−	0.22	0.28	0.593
rs714052	7q11.23	*MLXIPL*	+	0.13	3.27	0.0711
rs7706174	5q33.3	*TIMD4/HAVCR1*	−	0.17	0.78	0.284
rs873308	1p36.11	*TMEM57*	−	0.48	2.6	0.108
			**LDL Total Particles**			
rs10779835	1q42.13	*GALNT2*	+	0.61	0.3	0.586
rs10903129	1p36.11	*TMEM57*	−	0.47	2.39	0.3046
rs1363232	5q33.3	*TIMD4/HAVCR1*	−	0.32	0.065	0.419
rs4731702	7q32.2	*KLF14*	−	0.50	0.1	0.754
rs506585	2p24.1	*APOB*	−	0.22	2.47	0.037
rs6065906	20q13.12.B	*PLTP*	+	0.15	0.06	0.811
rs646776	1p13.3	*CELSR2/PSRC1/SPRT1*	−	0.20	12.43	0.001
rs6511720	19p13.2	*LDLR*	−	0.22	2.46	0.117
rs6982636	8q24.13	*TRIB1*	−	0.48	1.04	0.308
rs7307277	12q24.31.B	*CCDC92/DNAH10/ZNF664*	−	0.33	0.05	0.999
rs739461	19p13.11	*CILP2/PBX4/NCAN/SF4*	+	0.28	0.03	0.871
rs780094	2p23.3	*GCKR*	+	0.40	5.01	0.409
			**LDL Diameter**			
rs10808546	8q24.13	*TRIB1*	+	0.45	2.04	0.1537
rs10850435	12q24.11	*MVK/MMAB*	−	0.49	0.1	0.754
rs1800588	15q22.1	*LIPC*	+	0.22	4.49	0.034
rs1800775	16q13	*CETP*	+	0.45	3.46	0.064
rs2083637	8p21.3	*MADD/FOLH1/NR1H3*	+	0.27	2.76	0.097
rs2271293	16q22.1	*LCAT*	+	0.12	1.48	0.123
rs2957873	11p11.2–11p11.12	*MADD/FOLH1/NR1H3*	+	0.20	0.76	0.383
rs4846904	1q42.13	*GALNT2*	−	0.20	2.27	0.133
rs4939883	18q21.1	*LIPG*	−	0.21	0.53	0.439
rs6065906	20q13.12.B	*PLTP*	−	0.15	1.19	0.278
rs673548	2p24.1	*APOB*	+	0.22	0.2	0.537
rs7307277	12q24.31.B	*CCDC92/DNAH10/ZNF664*	+	0.33	1.11	0.137
rs7706174	5q33.3	*TIMD4/HAVCR1*	−	0.17	0.96	0.167
rs7777102	7q11.23	*MLXIPL*	+	0.21	4.48	0.035

Table S1. *Cont.*

Rs Number	Locus	*Gene*	± [a]	MAF in GOLDN	F-Value	*p*
**LDL Diameter**						
rs780094	2p23.3	*GCKR*	−	0.40	1.36	0.221
**Small HDL Concentration**						
rs1109166	16q22.1	*LCAT*	+	0.15	2.86	0.091
rs11974409	7q11.23	*MLXIPL*	−	0.26	4.87	0.028
rs1260326	2p23.3	*GCKR*	+	0.41	0.31	0.578
rs1532085	15q22.1	*LIPC*	−	0.37	3.92	0.048
rs2058804	12q24.11	*MVK/MMAB*	+	0.49	0	0.965
rs2163813	19p13.11	*CILP2/PBX4/NCAN/SF4*	+	0.72	0	0.989
rs3856637	3q22.3	*PCCB*	+	0.72	0.69	0.676
rs3924486	1p36.11	*TMEM57*	−	0.47	0.04	0.838
rs4752904	11p11.2–11p11.12	*MADD/FOLH1/NR1H3*	−	0.43	0.4	0.53
rs4847022	1q42.13	*GALNT2*	+	0.24	2.31	0.139
rs518181	11q23.3	*APOA1-A5*	+	0.36	5.97	0.015
rs6065906	20q13.12.B	*PLTP*	+	0.15	3.47	0.063
**Medium HDL Concentration**						
rs10779835	1q42.13	*GALNT2*	−	0.61	1.57	0.211
rs174537	11q12.2	*FADS1-3*	+	0.36	10.17	0.002
rs1800588	15q22.1	*LIPC*	−	0.22	0.3	0.587
rs2228603	19p13.11	*CILP2/PBX4/NCAN/SF4*	−	0.08	0	0.946
rs255052	16q22.1	*LCAT*	−	0.15	1.1	0.139
rs2909207	17q24.2.B	*WIPI1*	+	0.23	0.83	0.281
rs3905000	9q31.1	*ABCA1*	+	0.14	0.04	0.916
rs405509	19q13.32	*APOC1-APOE*	−	0.48	1.16	0.282
rs4073054	1q23.3	*APOA*	−	0.38	0.72	0.234
rs4704810	5q33.3	*TIMD4/HAVCR1*	+	0.43	0.92	0.337
**Large HDL Concentration**						
rs102275	11q12.2	*FADS1-3*	−	0.36	9.07	0.003
rs1800588	15q22.1	*LIPC*	+	0.22	13.20	0.0003
rs1800775	16q13	*CETP*	+	0.45	8.18	0.004
rs2058804	12q24.11	*MVK/MMAB*	−	0.49	0.64	0.422
rs2957873	11p11.2–11p11.12	*MADD/FOLH1/NR1H3*	+	0.20	0.24	0.622
rs331	8p21.3	*MADD/FOLH1/NR1H3*	+	0.26	0.27	0.61
rs4846904	1q42.13	*GALNT2*	−	0.20	4.46	0.035
rs4939883	18q21.1	*LIPG*	−	0.21	0.32	0.73
rs4986970	16q22.1	*LCAT*	−	0.04	0.34	0.559
rs6065906	20q13.12.B	*PLTP*	−	0.15	8.80	0.003
rs7307277	12q24.31.B	*CCDC92/DNAH10/ZNF664*	+	0.33	2.17	0.11
**HDL Total Particles**						
rs10779835	1q42.13	*GALNT2*	+	0.61	1.67	0.197
rs1109166	16q22.1	*LCAT*	+	0.15	4.17	0.042
rs1260326	2p23.3	*GCKR*	+	0.41	0.52	0.46

Table S1. *Cont.*

Rs Number	Locus	*Gene*	± [a]	MAF in GOLDN	F-Value	*p*
**HDL Total Particles**						
rs1818702	12q23.2	*intergenic, ASCL1, PAH*	−	0.31	1.17	0.895
rs2238675	19p13.11	*CILP2/PBX4/NCAN/SF4*	−	0.14	0.77	0.38
rs518181	11q23.3	*APOA1-A5*	+	0.36	0.49	0.486
rs6065906	20q13.12.B	*PLTP*	+	0.15	0.67	0.414
rs7499892	16q13	*CETP*	−	0.16	8.41	0.004
**HDL diameter**						
rs10850435	12q24.11	*MVK/MMAB*	−	0.49	0.9	0.344
rs1260326	2p23.3	*GCKR*	−	0.41	0.13	0.722
rs12610185	19p13.11	*CILP2/PBX4/NCAN/SF4*	+	0.08	1.14	0.285
rs1535	11q12.2	*FADS1-3*	−	0.36	8.13	0.004
rs1800588	15q22.1	*LIPC*	+	0.22	13.19	0.0003
rs1800775	16q13	*CETP*	+	0.45	4.68	0.031
rs2957873	11p11.2–11p11.12	*MADD/FOLH1/NR1H3*	+	0.20	0.2	0.65
rs331	8p21.3	*MADD/FOLH1/NR1H3*	+	0.26	2.23	0.126
rs4731702	7q32.2	*KLF14*	+	0.50	0.03	0.86
rs4846908	1q42.13	*GALNT2*	+	0.43	2.23	0.136
rs4939883	18q21.1	*LIPG*	−	0.21	0.37	0.499
rs4986970	16q22.1	*LCAT*	−	0.04	2.11	0.147
rs6065906	20q13.12.B	*PLTP*	−	0.15	4.72	0.03
rs7307277	12q24.31.B	*CCDC92/DNAH10/ZNF664*	+	0.33	1.37	0.124

[a] ± signifies the direction of effect in the original GWAS [1].

Table S2. Associations between a genetic risk score associated with baseline NMR measures, and the response of those NMR measures to a 3-week fenofibrate trial, stratified by baseline triglyceride levels.

**NMR Measure**	**GRS-Phenotype Associations with Fenofibrate Response**			
	**Baseline Fasting TG <150 mg/dL** ***n*** **= 544**		**Baseline Fasting TG ≥150 mg/dL** ***n*** **= 248**	
	**F**	***p***	**F**	***p***
Small VLDL concentration	0.22	0.64	0.27	0.61
Medium VLDL concentration	0.22	0.64	0.93	0.34
Large VLDL concentration	1.38	0.24	0.09	0.76
VLDL total particles	0.61	0.44	1.21	0.27
VLDL diameter	1.59	0.21	1.13	0.29
Small LDL concentration	0.62	0.43	0.10	0.75
Large LDL concentration	1.89	0.17	1.30	0.26
LDL total particles	0.05	0.82	0.22	0.64
LDL diameter	0.57	0.45	1.24	0.27
Small HDL concentration	1.41	0.24	0.22	0.64
Medium HDL concentration	5.64	0.02	0.77	0.39
Large HDL concentration	0.20	0.66	1.16	0.28
HDL total particles	0.97	0.33	0.80	0.37
HDL diameter	0.01	0.92	6.57	0.01

Reference

1. Chasman, D.I.; Paré, G.; Mora, S.; Hopewell, J.C.; Peloso, G.; Clarke, R.; Cupples, L.A.; Hamsten, A.; Kathiresan, S.; Mälarstig, A.; *et al*. Forty-three loci associated with plasma lipoprotein size, concentration, and cholesterol content in genome-wide analysis. *PLoS Genet*. **2009**, *5*, e1000730.

© 2014 by the authors; licensee MDPI, Basel, Switzerland. This article is an open access article distributed under the terms and conditions of the Creative Commons Attribution license (http://creativecommons.org/licenses/by/3.0/).
